# Low-Dose Salinomycin Alters Mitochondrial Function and Reprograms Global Metabolism in Burkitt Lymphoma

**DOI:** 10.3390/ijms26115125

**Published:** 2025-05-27

**Authors:** Aleksandra Zdanowicz, Oleksandr Ilchenko, Andrzej Ciechanowicz, Haoyu Chi, Marta Struga, Beata Pyrzynska

**Affiliations:** 1 Department of Biochemistry, Medical University of Warsaw, Banacha 1 Str., 02-097 Warsaw, Poland; 2 Doctoral School, Medical University of Warsaw, Zwirki i Wigury 81 Str., 02-091 Warsaw, Poland; 3 Department of Forest Genetics and Plant Physiology, Umeå Plant Science Centre, Swedish University of Agricultural Sciences, Skogsmarksgränd 17, 90183 Umea, Sweden; oleksandr.ilchenko@slu.se; 4 Department of Chemistry, Umeå University, Linnaeus väg 10B, 90187 Umea, Sweden; 5 Department of Regenerative Medicine, Center for Preclinical Research and Technology, Medical University of Warsaw, Banacha 1B Str., 02-097 Warsaw, Poland; 6 Department of Cell and Developmental Biology, University College London, Gower Street, London WC1E 6BT, UK; 7 Consortium for Mitochondrial Research, University College London, Gower Street, London WC1E 6BT, UK

**Keywords:** salinomycin, mitochondria, mitochondrial respiration, oxidative stress, metabolomics

## Abstract

Salinomycin (SAL), originally identified for its potent antibacterial properties, has recently garnered attention for its remarkable activity against a variety of cancer types. Beyond its direct cytotoxic effects on cancer cells, SAL can also enhance the efficacy of anti-CD20 immunotherapy in B-cell malignancies, both *in vitro* and *in vivo*. Despite these promising findings, the precise molecular mechanisms underlying SAL’s anticancer action remain poorly understood. Here, we demonstrate that even at low concentrations (0.25–0.5 mM), SAL disrupts mitochondrial membrane potential and induces oxidative stress in Burkitt lymphoma. Further investigations uncovered that SAL shifts cellular metabolism from mitochondrial respiration to aerobic glycolysis. Additionally, metabolomic profiling identified SAL-induced arginine depletion as a key metabolic alteration. These findings provide new insights into SAL’s multifaceted mechanisms of action and support its potential as an adjunctive therapy in cancer treatment.

## 1. Introduction

Polyether ionophores (cation carriers) constitute a significant class of natural compounds, capable of the selective binding and transporting of cations across cellular lipid membranes [1]. Among polyether ionophores, salinomycin (SAL) is the most extensively studied. Originally isolated from *Streptomyces albus*, it has been widely used as an anticoccidial and antibacterial agent against Gram-positive bacteria in veterinary medicine [2,3].

In a 2009 study, Gupta et al. were the first to demonstrate that SAL exhibited anticancer properties and a significantly greater potency and selectivity against breast cancer stem cells (CSCs) than conventional chemotherapeutic agents [4]. Since then, it has been demonstrated that SAL exhibits multi-targeted anticancer activity across diverse malignancies. In hepatocellular carcinoma, it induces cell cycle arrest in a cell line-dependent manner, causing G0/G1-phase arrest in HepG2 cells, while inducing G2/M-phase arrest in SMMC-7721 and BEL-7402 cells [5]. In colorectal cancer, SAL effectively suppresses tumor proliferation via inhibition of the Wnt/β-catenin signaling pathway [6]. In both prostate (PC3 cell line) and breast cancer cells (SKBR3 and MDAMB468), SAL modulates the mitochondrial dynamic and promotes mitophagy [7].

Further studies have revealed that SAL overcomes multidrug resistance in cisplatin-resistant ovarian cancer cells (A2780cis) by enhancing apoptosis through the upregulation of DR5 (death receptor-5), caspase-8, and FADD (Fas-associated protein with death domain) [8]. Additionally, SAL sensitizes radiation-treated breast cancer cells (Hs578T) by increasing DNA damage and inducing G2 arrest [9].

Our recent studies have shown that SAL not only possesses direct anticancer effects but also enhances the effectiveness of anti-CD20 immunotherapy by upregulating the surface CD20 antigen on malignant B-cells, as demonstrated in both *in vitro* and *in vivo* models [10]. B-cell malignancies—including diffuse large B-cell lymphoma (DLBCL), Burkitt lymphoma, follicular lymphoma, and high-grade B-cell lymphoma—are commonly treated with anti-CD20 monoclonal antibody therapy (e.g., rituximab, ofatumumab, obinutuzumab) in combination with chemotherapy [11]. However, treatment efficacy is often hindered by variable CD20 expression across different B-cell malignancy subtypes and among individual patients [12]. Notably, SAL shows promise for the upregulation of low CD20 levels in B-cell malignancies, offering a potential strategy to enhance target antigen expression and improve therapeutic outcomes [10]. While SAL demonstrates therapeutic potential in B-cell malignancies, its precise molecular mechanisms remain incompletely characterized.

Despite its diverse anticancer properties, concerns have been raised regarding its potential toxicity. In 2004, a 35-year-old patient reported experiencing nausea, tachycardia, and muscle pain following the accidental inhalation of SAL [13]. However, a pilot clinical trial involving patients with ovarian, head and neck, and metastatic breast cancers revealed that SAL administered intravenously at doses of 200–250 μg/kg every other day over three weeks led to the regression of metastatic lesions. Acute side effects were infrequent, and no serious long-term adverse effects were reported [14]. In contrast, subsequent studies have reported dose-dependent toxicity in mammals. For example, weight loss and sensory polyneuropathy were observed in mice at doses exceeding 5 mg/kg [15], while muscular atrophy and ataxia were reported in horses at doses above 0.6 µg/kg [16]. To overcome the potential adverse effects associated with SAL, numerous derivatives of SAL with improved anticancer properties have been synthesized [17,18], and are currently being tested in numerous preclinical models [19]. Boehmerle and Endres (2011) demonstrated that SAL-induced neuro- and myotoxicity is associated with increased cytosolic Na^+^ concentrations, which in turn trigger a rise in cytosolic Ca^2+^ levels [20]. Additionally, previous studies have demonstrated SAL’s ability to bind both monovalent (K^+^, Na^+^, Cs^+^) and divalent (Fe^2+^, Ca^2+^, Mg^2+^) metal cations [18], with a marked preference for monovalent species, particularly K^+^ and Na^+^ [17]. SAL forms complexes with these ions, facilitating their transport across lipid bilayer membranes [21]. SAL’s transport can occur through electroneutral, electrogenic or bio-mimetic mechanisms, depending on the surrounding environment—whether neutral, slightly alkaline, or acidic [17,22]. The ionophoric activity of SAL dysregulates the physiological Na^+^/K^+^ balance, alters the intracellular pH, and induces changes in osmotic pressure [17]. In breast CSCs, SAL has been shown to promote iron transport and storage within lysosomes, causing lysosomal membrane destabilization and ultimately triggering ferroptosis, a form of programmed cell death [18,23].

Recent studies have shown that SAL disrupts mitochondrial function in CSC [24], aligning with its well-established cationophoric activity across mitochondrial membranes [25]. In the MDA-MB-453 breast cancer cell line, treatment with 10 µM SAL for 48 h results in a reduction in MMP and a pronounced activation of caspases 3 and 7 [26]. In PC-3 prostate cancer cells, 48 h treatment with SAL (0.45–4 µM) promotes apoptosis through multiple mitochondrial-associated mechanisms, including enhanced ROS generation, MMP disruption, BAX mitochondrial translocation, the cytosolic accumulation of cytochrome c, PARP-1 cleavage, and caspase-3 activation [27]. In U2OS osteosarcoma cells, treatment with SAL for 12 and 24 h increases ROS production, which in turn activates AMPK signaling and induces autophagy [28]. Proteomic functional enrichment analysis of the breast CSC model (human mammary HMLER CD24low/CD44high), following a 48 h treatment with 0.5 µM SAL, revealed that SAL suppresses the expression of mitochondrial proteins associated with the TCA (tricarboxylic acid) cycle, ETC (electron transport chain), glycolysis, B-oxidation and glutaminolysis [24].

While Managò et al. [29] reported the rapid mitochondrial effects of SAL (10 μM) (within 0–90 min) in B-CLL cells, our study is the first to investigate the impact of prolonged (12–24 h), low-concentration SAL exposure on mitochondrial function and the global remodeling of Burkitt lymphoma cells’ metabolism.

## 2. Results

### 2.1. SAL Causes Mitochondrial Dysfunction and Induces Oxidative Stress in Burkitt Lymphoma Cells

**Figure 1:**
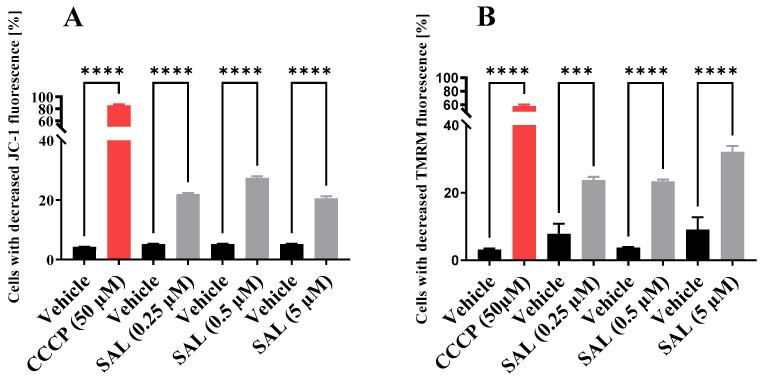
Salinomycin (SAL) reduces mitochondrial membrane potential (MMP) in live Raji cells. (**A**) MMP was assessed using the cationic carbocyanine dye JC-1 after 12 h of SAL treatment at concentrations of 0.25 µM, 0.5 µM, and 5 µM. (**B**) MMP was evaluated using tetramethylrhodamine methyl ester (TMRM) following 36 h of SAL treatment at the same concentrations. A positive control was included by treating cells with 50 µM carbonyl cyanide 3-chlorophenylhydrazone (CCCP) for 1 h. Data are expressed as mean ± SD from three experiments (*n* = 3), and statistical significance was assessed using one-way ANOVA (*** *p*  <  0.001, **** *p*  <  0.0001).

To assess the effects of SAL on mitochondrial function in Burkitt lymphoma, we treated Raji cells with 0.25 μM, 0.5 μM, or 5 μM SAL for 12 and 36 h. Staining with membrane potential-sensitive fluorescent probes (JC-1 and TMRM), followed by flow cytometry analysis, revealed a significant reduction in mitochondrial membrane potential (MMP) following SAL treatment compared to matched controls (Figure 1A,B and Supplementary Figures S1 and S2). The MMP generated by mitochondrial complexes I, III, and IV reflects the mitochondrion’s capacity for ATP production [30]. Based on this, we hypothesize that SAL induces mitochondrial dysfunction.

**Figure 2:**
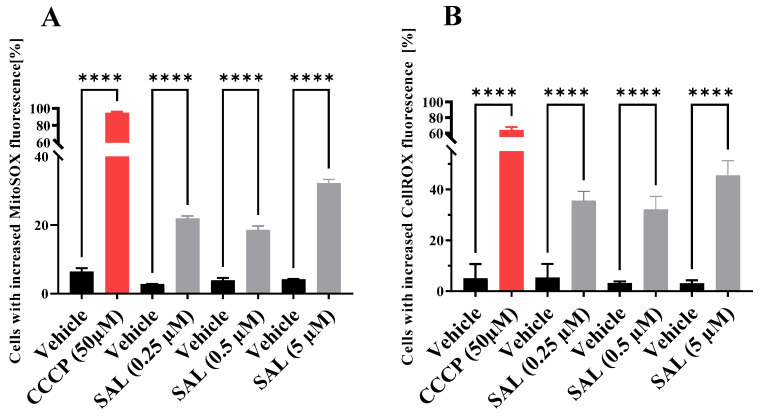
SAL induces the generation of reactive oxygen species (ROS) in Raji cells. (**A**) Mitochondrial superoxide production was detected using MitoSOX dye following 36 h of SAL treatment at concentrations of 0.25 µM, 0.5 µM, and 5 µM. (**B**) Total ROS levels were assessed using CellROX Green reagent after 48 h of SAL treatment at concentrations of 0.25 µM and 0.5 µM. As a positive control, cells were treated with 50 µM CCCP for 1 h. Results are expressed as mean ± SD from three experiments (*n* = 3), and statistical significance was evaluated using one-way ANOVA (**** *p*  <  0.0001).

Given that mitochondrial dysfunction can influence respiratory chain activity and the production of mitochondrial reactive oxygen species (mtROS), we evaluated mtROS levels using MitoSOX Red, a fluorogenic dye that selectively detects superoxide in live mitochondria. Quantitative analysis using flow cytometry revealed a significant increase in the generation of mitochondrial superoxide following treatment with 0.25 μM, 0.5 μM, and 5 μM of SAL (Figure 2A and Supplementary Figure S3). The continuous elevation in mtROS disrupts redox homeostasis, leading to an imbalance between ROS production and the cellular antioxidant defense system [31,32]. To quantify this oxidative stress, we utilized CellROX Green reagent, a fluorogenic probe for live-cell ROS detection. Treatment with 0.25 and 0.5 μM SAL resulted in a significant increase in oxidative stress compared to vehicle-treated controls (Figure 2B and Supplementary Figure S4).

### 2.2. SAL Suppresses Mitochondrial Respiration and Glycolytic Activity

**Figure 3:**
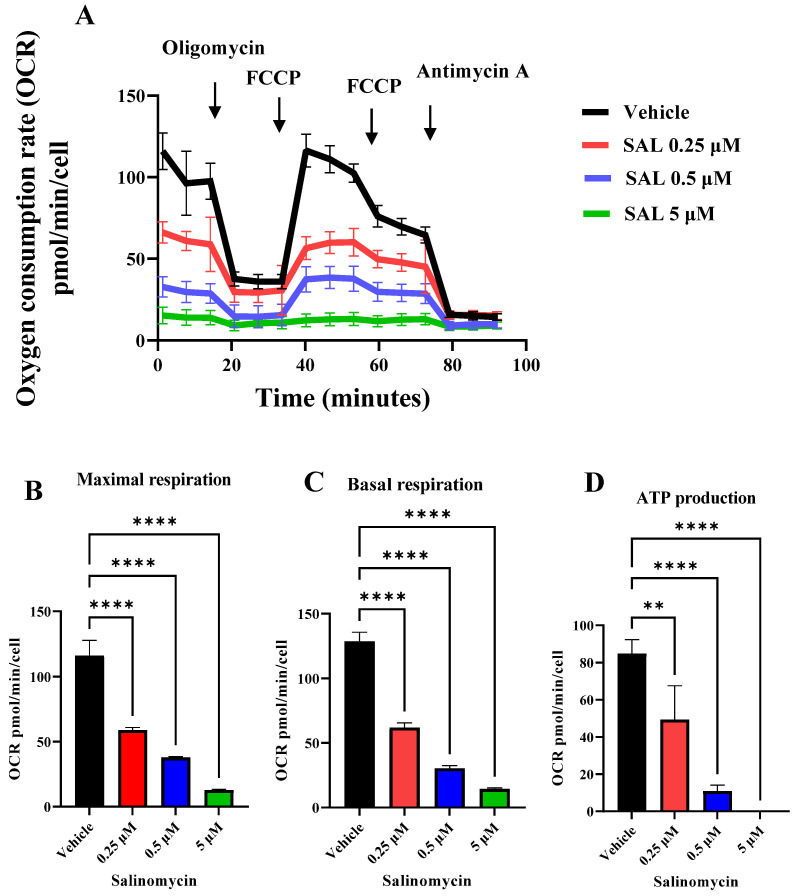
SAL suppresses mitochondrial respiration in Raji cells. (**A**) Oxygen consumption rate (OCR) was measured using a Seahorse XFe96 analyzer, following 24 h-long treatment with 0.25 µM, 0.5 µM, or 5 µM SAL. OCR was assessed under basal conditions and after the sequential addition of oligomycin (2.5 µM), carbonyl cyanide-p-trifluoromethoxyphenylhydrazone (FCCP, 1 µM and 1.5 µM), and antimycin A (2.5 µM). The data concerning maximal respiration (**B**), basal respiration (**C**), and ATP production (**D**) have been extracted from the analysis presented in (**A**). Data were normalized to cell number and represent the mean ± SD from experiments (*n* = 3). Statistical analysis was performed using one-way ANOVA (** *p*  <  0.01, **** *p*  <  0.0001).

To directly evaluate mitochondrial dysfunction, we measured oxygen consumption rates (OCRs) using the Seahorse XFe96 extracellular flux analyzer. After 24 h of SAL treatment, both basal and maximal respiratory capacities were significantly reduced (Figure 3A–C), accompanied by a substantial decrease in ATP production (Figure 3D). These findings indicate a profound impairment of mitochondrial oxidative phosphorylation even at the lowest concentration of SAL (0.25 μM).

**Figure 4:**
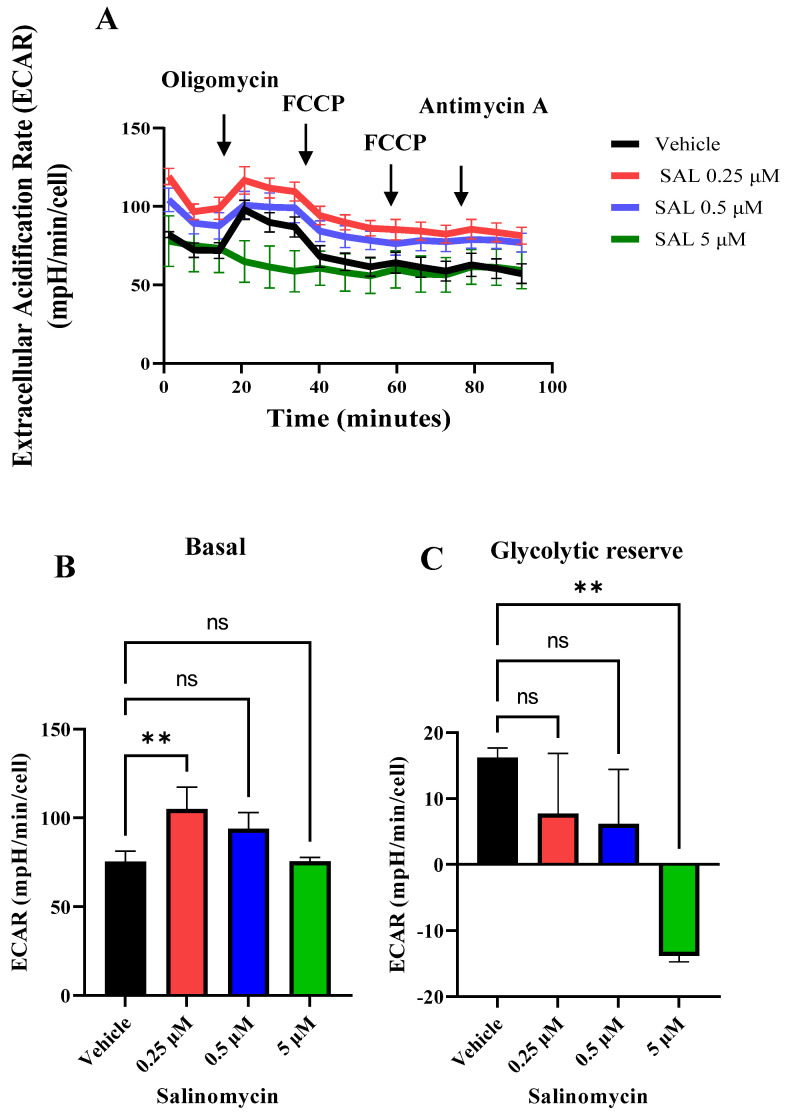
SAL reduces glycolytic activity in Raji cells. (**A**) Extracellular acidification rate (ECAR) was measured using the Seahorse XFe96 analyzer following treatment with 0.25 µM, 0.5 µM, and 5 µM SAL under basal conditions and after sequential injections of oligomycin (2.5 µM), FCCP (1 µM and 1.5 µM), and antimycin A (2.5 µM). (**B**,**C**) Quantification of basal ECAR (**B**) and glycolytic reserve in response to oligomycin (**C**) was extracted from the ECAR profiles shown in (**A**). Data were normalized to cell number and represent the mean ± SD from three experiments (*n* = 3). Statistical analysis was performed using one-way ANOVA (** *p*  <  0.01, ns indicates non-significant differences (*p* > 0.05)).

To examine the impact of SAL on glycolytic flux, we measured the extracellular acidification rate (ECAR). Notably, 24 h treatment with SAL led to significant metabolic reprogramming, as evidenced by a marked increase in basal ECAR at a concentration of 0.25 μM. While the glycolytic reserve remained unchanged at lower concentrations (0.25 μM and 0.5 μM), it was decreased at 5 μM (Figure 4A–C). These findings suggest that SAL suppresses oxidative phosphorylation, while enhancing glycolytic activity, in Burkitt lymphoma cells.

### 2.3. SAL Reprograms Burkitt Lymphoma Metabolism

Mitochondria play a vital role in cancer cell adaptation and metabolic regulation, especially under stressful conditions [31,33]. To investigate the metabolic alterations associated with SAL-induced mitochondrial dysfunction, we conducted untargeted metabolomic profiling using a SolariX 2xR 7T FT-ICR MS on cells treated with 0.25 µM SAL for 24 h.

This analysis detected 9290 metabolic features—5492 in positive ion mode and 3798 in negative ion mode. Among these, 698 metabolites were annotated (425 in positive mode and 273 in negative mode). After filtering out duplicates and excluding metabolites present in fewer than 30% of samples, 93 metabolites (76 from positive mode and 17 from negative mode) were retained for further analysis. Although different normalization methods were applied to the dataset, the subsequent analysis focused solely on the Pareto-normalized data. The Pareto-normalized dataset exhibits a kurtosis of 2.65 and a skewness of −0.49, with a coefficient of determination (R^2^) fitted to a Gaussian distribution of 0.884 and 2.15% of significant *p*-values from the one-sample Kolmogorov–Smirnov test.

**Figure 5:**
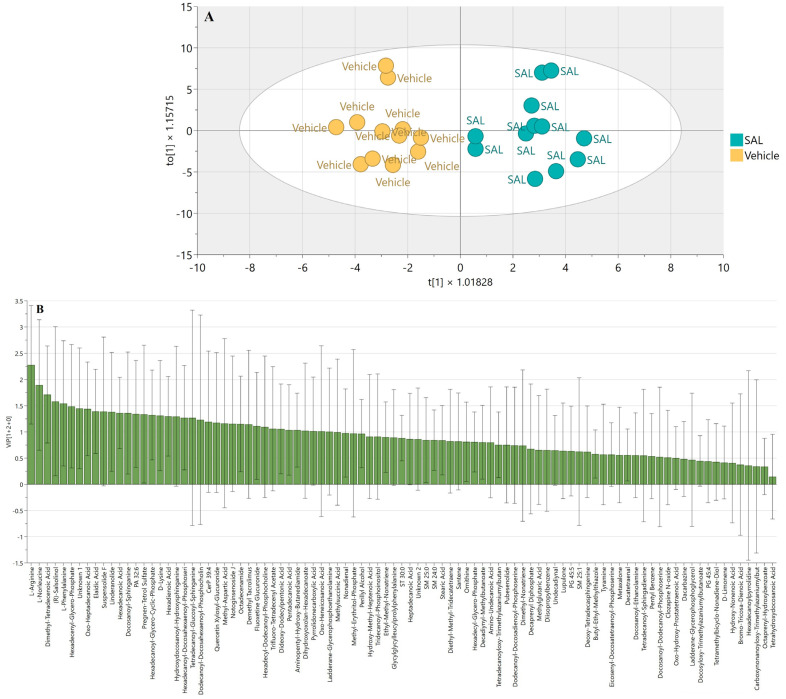
Untargeted metabolomic profiling of Raji cells treated with 0.25 µM SAL, analyzed using SolariX 2xT 7T. (**A**) OPLS-DA (Orthogonal Projections to Latent Structures–Discriminant Analysis) score plot R2X = 0.104, R2Y = 0.878, Q2 = 0.476; CV-ANOVA *p* = 0.0399. (**B**) OPLS-derived VIP (Variable Importance in Projection) score plot.

An OPLS-DA (Orthogonal Partial Least Squares Discriminant Analysis) model, along with PCA (Principal Component Analysis), was applied to compare the metabolomic profiles of SAL-treated and vehicle-treated cells (Figure 5A and Supplementary Figures S5 and S6). To pinpoint the most significant metabolic alterations, we examined the Variable Importance in Projection (VIP) scores derived from the OPLS model. This analysis highlighted marked changes in the levels of L-arginine and L-norleucine following SAL treatment (Figure 5B).

**Figure 6:**
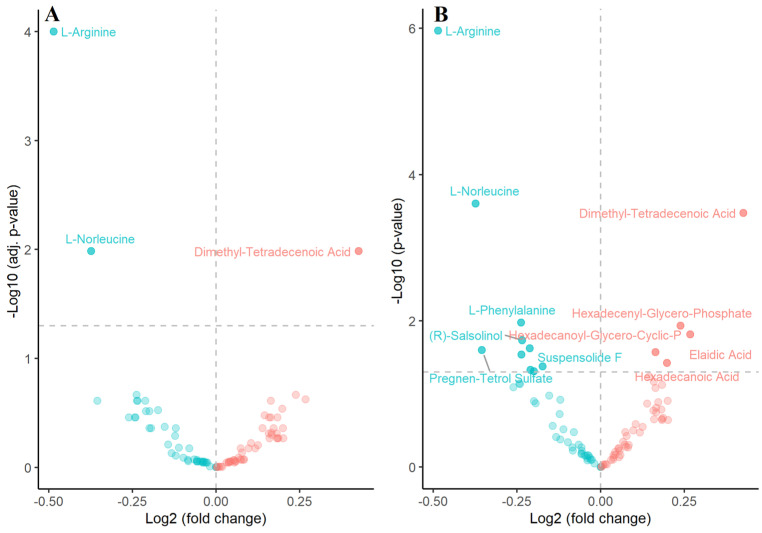
Metabolome of SAL-treated Raji cells. Volcano plots display differentially regulated metabolites, with the most significant ones marked with annotations, based on (**A**) FDR-adjusted *p*-values (*t*-test *p* = 0.05) and (**B**) unadjusted *p*-values (*t*-test *p* = 0.05). Blue represents metabolites that are lower in the SAL-treated group, while red denotes metabolites that are higher relative to the vehicle group. Horizontal dashed line indicates *p* = 0.05.

**Figure 7:**
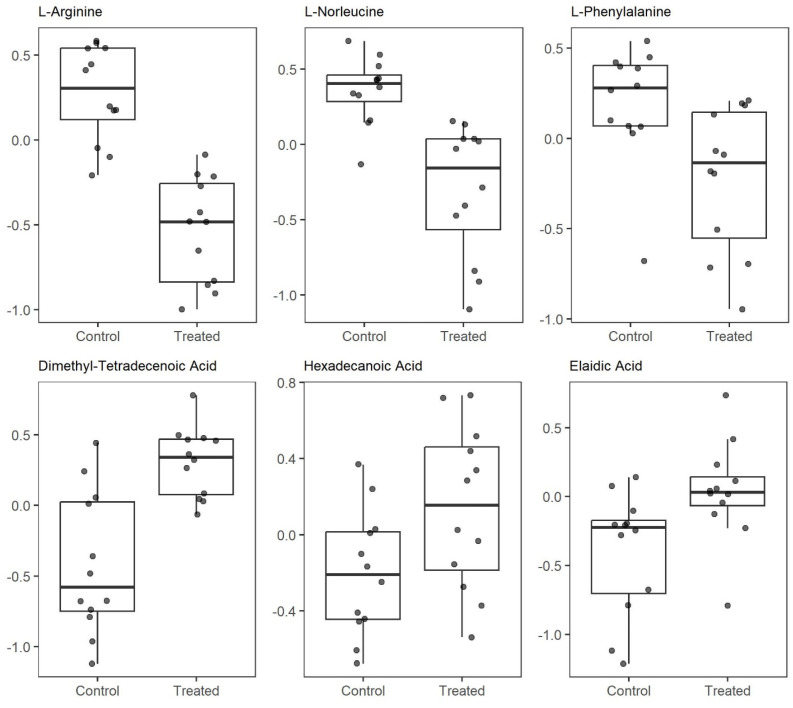
Levels of metabolites increased or decreased following treatment with 0.25 µM SAL (*t*-test *p* = 0.05).

A pairwise comparison of normalized metabolomic data between SAL-treated and vehicle-treated samples was performed using Student’s *t*-test (*p* < 0.05). The results were visualized using volcano plots (Figure 6A,B), highlighting statistically significant metabolites. This analysis revealed notable alterations in the levels of several metabolites following SAL treatment, including a marked decrease in L-arginine (*t*-test, *p* = 0.000001), L-norleucine (*p* = 0.0002), and L-phenylalanine (*p* = 0.01) (Figure 7 and Supplementary Figure S7). In contrast, SAL treatment resulted in a significant increase in dimethyl-tetradecenoic acid (*p* = 0.0003), hexadecenoic acid (*p* = 0.03), and elaidic acid (*p* = 0.015).

**Figure 8:**
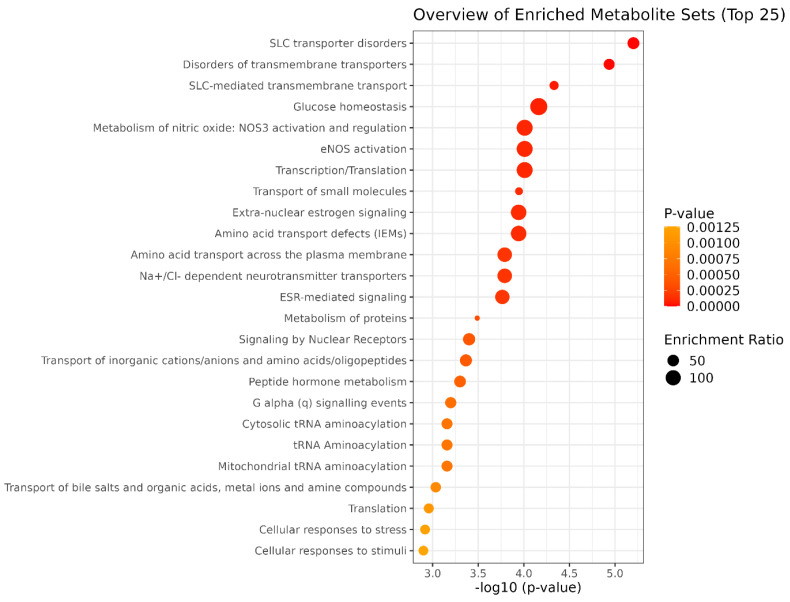
Enrichment analysis of metabolic pathways after SAL’s administration using Metaboanalyst 6.0. The top 25 enriched pathways are displayed based on *p*-value and enrichment ratio.

To identify metabolic pathways altered in response to SAL’s administration, we performed a metabolite set enrichment analysis (MSEA) using MetaboAnalyst 6.0 (Figure 8). The analysis revealed significant changes in pathways related to solute carrier (SLC)-mediated transmembrane transport and glucose homeostasis. Of note was that the revealed changes in glucose homeostasis aligned with the changes in glycolysis status, previously detected by the Seahorse extracellular flux analyzer.

## 3. Discussion

SAL not only demonstrates direct anticancer properties, but it has also recently been shown to enhance anti-CD20 immunotherapy in both *in vitro* and *in vivo* models of B-cell malignancies [10]. These findings underscore its potential for advancing into clinical trials and eventual therapeutic applications. Building on this, our study seeks to further elucidate the molecular mechanisms driving SAL’s anticancer effects in Burkitt lymphoma. A more comprehensive and precise understanding of these mechanisms could substantially improve SAL’s prospects for clinical success.

In the present study, we investigated the effects of a non-toxic concentration of SAL on mitochondrial function and metabolomic profiles in Burkitt lymphoma. Specifically, we focused on SAL’s impact on MMP and mitochondrial respiration and its role in reprogramming cellular metabolism. The observed decrease in MMP in Burkitt lymphoma cells following 0.25 µM SAL treatment (Figure 1A,B and Supplementary Figure S1) is consistent with findings in other cancer models. For instance, SAL at 5 µM was reported to reduce MMP in retinoblastoma RB383 cells [34], and at 1.33 µM in human prostate cancer cells (PC-3) [27]. Similarly, a 10 µM concentration of SAL decreased MMP in PC-3, SKBR3, and MDA-MB-468 cancer cell lines [7]. In the melanoma SK-Mel-19 cell line, treatment with 1 μM SAL for 12 and 24 h resulted in a reduction in MMP levels [35].

Our data indicate that SAL induces a sustained reduction in MMP, a phenomenon whose pathological significance extends beyond simple ATP deficiency. Furthermore, MMP plays a crucial role in maintaining mitochondrial homeostasis and provides the electrochemical gradient necessary for ion transport across the mitochondrial membrane [30]. As highlighted by Zorova et al., prolonged MMP suppression primarily affects ROS levels, potentially leading to oxidative stress [30]. Consistent with this, our findings show that SAL induces oxidative stress (Figure 2B and Supplementary Figure S4) in line with previous reports in various cancer models. For instance, Xipell et al. reported that SAL promotes global ROS generation in glioma cells (GSC11 and SF188) at a concentration of 0.1 µM [36]. Similarly, Yu et al. reported increased ROS levels in U87MG cells treated with 4 µM SAL [37]. A 6 h treatment with SAL at concentrations of 1.89 µM in A549 lung cancer cells and 1.22 µM in MDA-MB-231 breast cancer cells resulted in only a marginal, statistically insignificant increase in oxidative stress [38].

In our study, SAL treatment led to a marked increase in mtROS levels (Figure 2A and Supplementary Figure S3). Previous research using 10 µM SAL in B-CLL cells showed unchanged mtROS levels during over 60 min of exposure [29]. The observed increase in mtROS levels may be attributed to changes in mitochondrial respiration, as ROS production is closely linked to electron transport chain activity and oxygen consumption. While it is well established that an elevated MMP is associated with an increased production of mtROS, a reduction in MMP can lead to decreased ROS levels, primarily due to diminished activity of mitochondrial Complexes I and III, which are the main sites of mtROS generation [39,40]. Conversely, in pathological conditions such as hypoxia, a reduced MMP has been linked to elevated mtROS production, contributing to excessive overall oxidative stress [41]. This is further supported by the alterations in OCR detected following SAL treatment (Figure 3A), consistent with previous reports in retinoblastoma RB383 cells [34] and HMLE-Twist cells [29].

Our data demonstrate that SAL-induced mitochondrial dysfunction, characterized by a reduction in MMP (Figure 1A,B) and OCR (Figure 3), appears to elicit a compensatory upregulation of glycolytic metabolism. This is supported by a significant increase in the basal ECAR following treatment with 0.25 µM SAL (Figure 4A,B). Notably, this metabolic adaptation differs from observations in retinoblastoma RB383 cells, where SAL treatment did not result in a significant change in basal ECAR [34]. In HMLER CD24⁻ cells, SAL treatment resulted in a greater reliance on glycolytic activity compared to mitochondrial respiration [24]. Together, these findings suggest that SAL treatment promotes metabolic reprogramming toward enhanced aerobic glycolysis as a primary source of ATP production.

Our metabolomic analysis revealed that a low concentration of SAL led to a decrease in L-arginine (Figure 7), a metabolite synthesized from citrulline and aspartate via arginosuccinate synthase 1 (ASS1) as part of the urea cycle [42]. Reduced arginine levels have been linked to mitochondrial dysfunction, primarily due to impaired oxidative phosphorylation (OXPHOS) and decreased ATP production in ASS1-deficient breast cancer [43]. In MDA-MB-231 breast adenocarcinoma cells, arginine deprivation disrupts both mitochondrial respiration and glycolytic flux, leading to extensive metabolic reprogramming [44]. This response is characterized by dysregulated ROS levels and altered acetyl-CoA production. Notably, cells with impaired mitochondrial function exhibit resistance to the effects of arginine starvation, highlighting the critical role of mitochondrial function in mediating this metabolic vulnerability [44]. In ASS1-deficient leiomyosarcoma (SKLMS1, SKUT1) and melanoma (SKMEL2) cells, arginine deprivation suppresses the Warburg effect while promoting OXPHOS [45]. In a 2020 study, Brashears et al. demonstrated that the cellular response to arginine starvation in sarcomas is mediated by ERK’s upregulation and activation of the MYC signaling pathway [46].

Arginine deprivation has been associated with several forms of cell death, including caspase-dependent and -independent apoptosis, autophagic cell death, and necroptosis [42]. These mechanisms have been reported across a range of malignancies, including ovarian cancer [47], sarcoma [48], melanoma [49], and lymphoma [50]. As a result, arginine deprivation has emerged as a promising and advanced therapeutic strategy in cancer treatment. Several approaches have been investigated to achieve arginine depletion, including dietary restriction, inhibition of arginine transport, and enzymatic therapies such as recombinant human arginase and arginine deiminase [51]. Among these, human recombinant arginase I (PEG-BCT-100) and pegylated arginine deiminase (ADI-PEG20) are currently in clinical trials and have shown success in effectively depleting arginine levels in cancer cells. To date, more than 20 clinical trials have evaluated ADI-PEG20 as a monotherapy or in combination with other treatments across over 12 cancer types, including glioblastoma, melanoma, non-small cell lung cancer, mesothelioma, acute myeloid leukemia, pancreatic cancer, hepatocellular carcinoma, breast cancer, and uveal melanoma [42].

In summary, our findings demonstrate that SAL, even at low concentrations, diminishes MMP, elevates mtROS, and induces oxidative stress in Burkitt lymphoma cells. Furthermore, SAL disrupts mitochondrial respiration, boosts glycolytic activity, and decreases intracellular arginine levels. Furthermore, metabolic pathway enrichment analysis (Figure 8) revealed significant alterations in SLC-mediated transmembrane transport, which is an expected outcome given SAL’s ability to influence ion transport via biological membranes.

However, this study has certain limitations, including the use of a single cell line and the absence of validation in additional lymphoma cell lines or primary patient samples. These factors may limit the generalizability of the results and underscore the need for further investigations in more physiologically relevant models to confirm and extend these findings.

## 4. Materials and Methods

### 4.1. Cell Line and Chemicals

The Burkitt lymphoma Raji cell line (RRID:CVCL_0511) was maintained in RPMI 1640 medium (Corning, NY, USA; cat. #10-040-CVR) supplemented with 10% fetal bovine serum (FBS; Fisher Scientific, Waltham, MA, USA; cat. #SH3007203), 100 U/mL penicillin, and 100 μg/mL streptomycin at 37 °C in a humidified 5% CO_2_ atmosphere. The cell line was routinely tested and confirmed to be mycoplasma-free. Salinomycin (sodium salt) was obtained from Sigma-Aldrich, St. Louis, MO, USA (cat. #S4526) and dissolved in methanol.

### 4.2. Mitochondrial Membrane Potential (MMP) Assessment

Cells (1.5 × 10^5^ per well) were seeded in 12-well plates and treated with either methanol (control vehicle) or salinomycin (SAL; 0.25 μM, 0.5 μM, or 5 μM). After 12 or 36 h of treatment, cells were harvested by centrifugation (1300 rpm, 5 min, 4 °C) and washed with phosphate-buffered saline (PBS).

MMP was evaluated using either the MitoPT JC-1 Assay Kit (ImmunoChemistry Technologies, Davis, CA, USA, cat. #6261) or the MitoProbe TMRM Assay Kit (Invitrogen, Waltham, MA, USA, cat. #M20036), following the manufacturer’s instructions. As a positive control, cells were treated with 50 µM carbonyl cyanide 3-chlorophenylhydrazone (CCCP) for 60 min. Cells were co-stained with either JC-1 or TMRM and Zombie-NIR Dye (BioLegend, San Diego, CA, USA, cat. #423106) for 30 min at 37 °C in the dark, washed with PBS, and live cells were subsequently analyzed by flow cytometry using a FACS Verse instrument (Becton Dickinson, NJ, USA). In both the MitoPT JC-1 and MitoProbe TMRM assays, the percentage of live cells (Zombie-NIR-negative) displaying reduced fluorescence in the PE (phycoerythrin) channel was assessed.

### 4.3. Assessment of Mitochondrial Superoxide and Global Oxidative Stress

Cells (1.5 × 10^5^ per well) were plated in 12-well plates and treated with either salinomycin (Sal; 0.25 μM, 0.5 μM, or 5 μM) or the control vehicle. Following treatment periods of 36 or 48 h, cells were harvested by centrifugation (1300 rpm, 5 min, 4 °C) and washed with ice-cold PBS. Mitochondrial superoxide production was measured using MitoSOX Red mitochondrial superoxide indicator (Invitrogen, Waltham, MA, USA, cat. #M36008), while general oxidative stress was assessed with CellROX Green Reagent (Invitrogen, Waltham, MA, USA, cat. #C10492), according to the manufacturer’s protocols. As a positive control, cells were treated with 50 µM CCCP for 60 min. Following a 30 min co-staining with Zombie-NIR Dye (BioLegend, San Diego, USA, cat. #423106) under light-protected conditions, samples were immediately analyzed by flow cytometry using a FACS Verse instrument (Becton Dickinson, New Jersey, USA). In the MitoSOX Red assay, the percentage of live cells (Zombie-NIR-negative) exhibiting increased fluorescence in the PE channel was measured. For the CellROX Green Reagent assay, the percentage of live cells (Zombie-NIR-negative) showing elevated fluorescence in the FITC (fluorescein) channel was determined.

### 4.4. Measurement of Cellular Bioenergetics Using Seahorse XFe96 Extracellular Flux Analyzer

Cellular respiration and glycolysis were measured using the Seahorse XFe96 extracellular flux analyzer (Agilent, Santa Clara, CA, USA) with the XF Cell Mito Stress Test Kit (Agilent, Santa Clara, CA, USA, cat. #103015-100). Assays were conducted in Seahorse XF DMEM Medium (pH 7.4; Agilent), supplemented with glucose (1 mM; Gibco, Waltham, MA, USA, cat. #A24940-01), L-glutamine (1 mM; Gibco, Waltham, MA, cat. #25030), and sodium pyruvate (1 mM; Sigma, cat. #S8636). Non-adherent Raji cells (5 × 10^4^ cells/well) were seeded onto Cell-Tak (Corning, Corning, NY, USA, cat. #354240)-coated XF96 microplates (Agilent, Santa Clara, CA, USA, cat. #102416-100) to ensure adhesion and incubated for 30 min at 37 °C in a CO_2_-free incubator before the assay’s initiation. Following baseline measurements, the following compounds were sequentially injected: oligomycin (2.5 µM; ATP synthase inhibitor), carbonyl cyanide-p-trifluoromethoxyphenylhydrazone (FCCP, 1 µM followed by 1.5 µM; mitochondrial uncoupler to assess maximal respiration), and antimycin A (2.5 µM; Complex III inhibitor). The initial low concentration of FCCP helps determine the minimal dose required to achieve a maximal OCR, while the subsequent higher concentration confirms whether maximal respiration has been fully reached. Data were analyzed using the XF Cell Mito Stress Test Report Generator (Agilent, Santa Clara, USA). Post-assay normalization was performed by staining cells with Hoechst 33342 (5 µM; Thermo Scientific, Waltham, MA, USA, cat. #62249) for 30 min. Nuclei were quantified using the ImageXpress imaging system (Molecular Devices, San Jose, CA, USA) to determine cell counts per well, and all Seahorse measurements were normalized accordingly.

### 4.5. Metabolomics Sample Preparation

Cells were normalized by count and lysed via a freeze–thaw cycle, followed by the addition of 100 μL of ice-cold (−20 °C) methanol (LC-MS hypergrade, Merck, Darmstadt, Germany). After centrifugation (18,000× *g*, −10 °C, 30 min), 80 μL of the supernatant was lyophilized at room temperature using an Eppendorf concentrator. The dried residue was reconstituted immediately prior to injection in a 50:50 (*v*/*v*) mixture of ddH_2_O and acetonitrile (ACN) containing 0.1% formic acid.

Untargeted metabolomic profiling was conducted using a SolariX 2xR 7T FT-ICR MS (ultra-high-resolution Fourier-transform ion cyclotron resonance mass spectrometer) equipped with an electrospray ionization (ESI) source in direct injection mode. Samples were infused at a flow rate of 300 μL/h in both positive and negative ionization modes.

Key instrument parameters:

Ion accumulation time: 0.03 s;

Dry gas flow: 4.0 L/min;

Drying temperature: 200 °C;

Capillary voltage: 4500 V (positive mode)/3500 V (negative mode).

Mass spectra were acquired and processed using the T-Rex 2D algorithm (MRMS single spectra) within MetaboScape 5.0 software (Bruker, Billerica, MA, USA).

#### 4.5.1. Sample Overview

The dataset, which comprised metabolomic profiles, was acquired in both positive and negative ionization modes using MetaboScape 5.0 software. Samples included the control group (vehicle-treated cells) and the treatment group (cells treated with 0.25 μM salinomycin for 24 h).

The experimental design consisted of 24 total samples:

12 control samples (6 independent replicates × 2 technical replicates);

12 treated samples (6 independent replicates × 2 technical replicates).

#### 4.5.2. Data Treatment

To ensure a high data quality and analytical robustness, the following treatment steps were applied (Table 1):

1. Missing Value Imputation

Sparse data (zero values) were imputed to maintain dataset completeness and enhance statistical reliability.

2. Data Transformation

Variables were mathematically transformed to approximate normal distributions and reduce skewness, ensuring compliance with parametric test assumptions.

3. Data Centering

Values were adjusted to a common scale using min–max normalization ([0, 1] range) to facilitate direct comparison across variables and samples.

4. Data Normalization

Systematic technical variations were corrected using normalization methods to isolate biologically relevant patterns.

### 4.6. Statistical Analysis

#### 4.6.1. Descriptive Statistical Analysis

Comprehensive quality control was performed using descriptive statistical analysis to assess data integrity and the effectiveness of treatments. The following metrics were calculated:

- Distribution characteristics: kurtosis and skewness—evaluated deviations from normality;
- Gaussian function fit R^2^—assessed the goodness-of-fit to a normal distribution;
- One-sample Kolmogorov–Smirnov test—statistically tested normality assumptions.

#### 4.6.2. Univariate Statistical Analysis

- One-sample test Kolmogorov–Smirnov—evaluated distribution normality;
- Student’s *t*-test—applied to normally distributed data;
- Mann–Whitney U test—used for non-normally distributed data or in the presence of outliers;
- Multiple comparison correction: Benjamini–Hochberg procedure—controlled the false discovery rate (FDR).

#### 4.6.3. Multivariate Statistical Analysis

To explore patterns and group separations in the metabolomic data, complementary multivariate approaches were applied:

1. Unsupervised Analysis:

- Principal Component Analysis (PCA): provided an initial unsupervised overview of data structure and helped identify outliers.

2. Supervised Modeling:

- Orthogonal Partial Least Squares Discriminant Analysis (OPLS-DA): used to enhance group separation and identify discriminant metabolites;
- Model optimization: performed through iterative component selection;
- Validation: conducted using leave-one-out cross-validation (LOOCV);
- Significance testing: assessed via cross-validated ANOVA (CV-ANOVA), with a *p* < 0.05 considered significant.

3. Model Quality Assessment:

- Permutation testing: evaluated potential overfitting.

4. Calculated model parameters:

- R^2^X/R^2^Y: goodness of fit of the model;
- Q^2^: goodness of prediction of the model.

## Figures and Tables

**Table 1:** Summary of data treatment steps.

Step	Type	Method	Equation
1	Missing Value Imputation	Half-minimum	$x_{ij}=\frac{{\bar{x}}_{i}}{2}$
2	Data Transformation	Log_10_	${\tilde{x}}_{ij}={log}_{10}(x_{ij})$
3	Data Centering	Min–max centering	${\hat{x}}_{ij}=\frac{{\tilde{x}}_{ij}-{\tilde{x}}_{j\;min}\;}{{\tilde{x}}_{j\;max}-{\tilde{x}}_{j\;min}}$
4	Data Normalization	Pareto	${\tilde{x}}_{ij}=\frac{\left({\hat{x}}_{ij}-{\bar{x}}_{i}\right)}{\surd s_{i}}$

## Data Availability

Data are contained within the article and Supplementary Materials.

## Supplementary Materials

The following supporting information can be downloaded at: https://www.mdpi.com/article/10.3390/ijms26115125/s1.

## Abbreviations

The following abbreviations are used in this manuscript:

ASS1	Arginosuccinate synthase 1
CCCP	Carbonyl cyanide 3-chlorophenylhydrazone
CSC	Cancer stem cell
CV-ANOVA	Cross-validated ANOVA
DLBCL	Diffuse large B-cell lymphoma
DR5	Death receptor-5
ECAR	Extracellular acidification rate
ETC	Electron transport chain
FADD	Fas-associated protein with death domain
FCCP	Carbonyl cyanide-p-trifluoromethoxyphenylhydrazone
LOOCV	Leave-one-out cross-validation
MMP	Mitochondrial membrane potential
MSEA	Metabolite set enrichment analysis
mtROS	Mitochondrial reactive oxygen species
OCR	Oxygen consumption rates
OPLS-DA	Orthogonal Partial Least Squares Discriminant Analysis
OXPHOS	Oxidative phosphorylation
PCA	Principal Component Analysis
SAL	Salinomycin
SLC	Solute carrier
TCA	Tricarboxylic acid
TMRM	Tetramethylrhodamine methyl ester
VIP	Variable Importance in Projection
