# Supplementary material for: Low-Dose Salinomycin Alters Mitochondrial Function and Reprograms Global Metabolism in Burkitt Lymphoma
Source: Int J Mol Sci. 2025 May 27;26(11):5125. doi: 10.3390/ijms26115125 (PMC12155122; doi:10.3390/ijms26115125)
Supplement: Supplementary file 1 [file ijms-26-05125-s001.zip › ijms-3636543-supplementary.pdf]

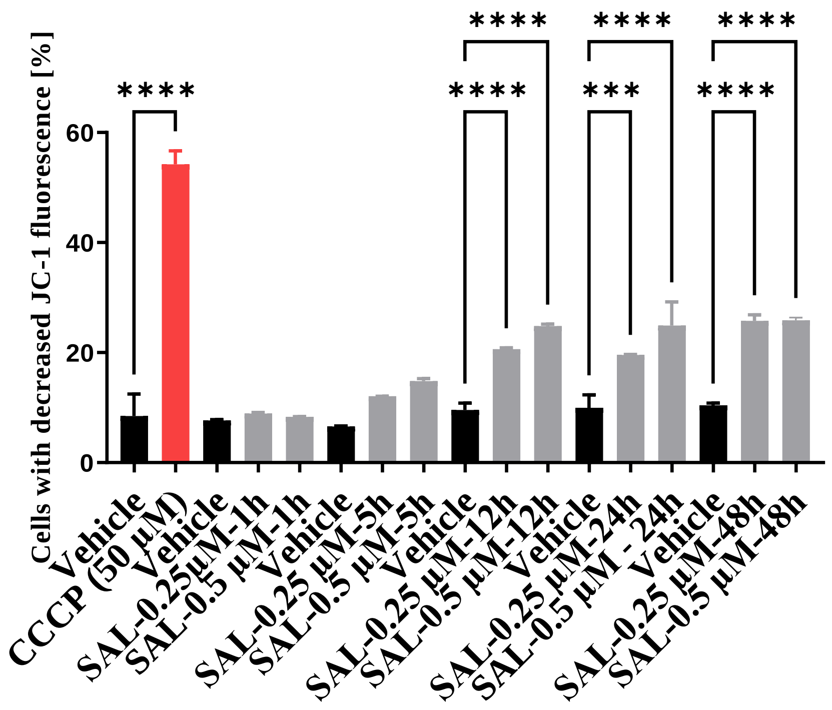

Supplementary Figure S1: Time-dependent changes in MMP following SAL treatment, visualized using JC-1 staining.

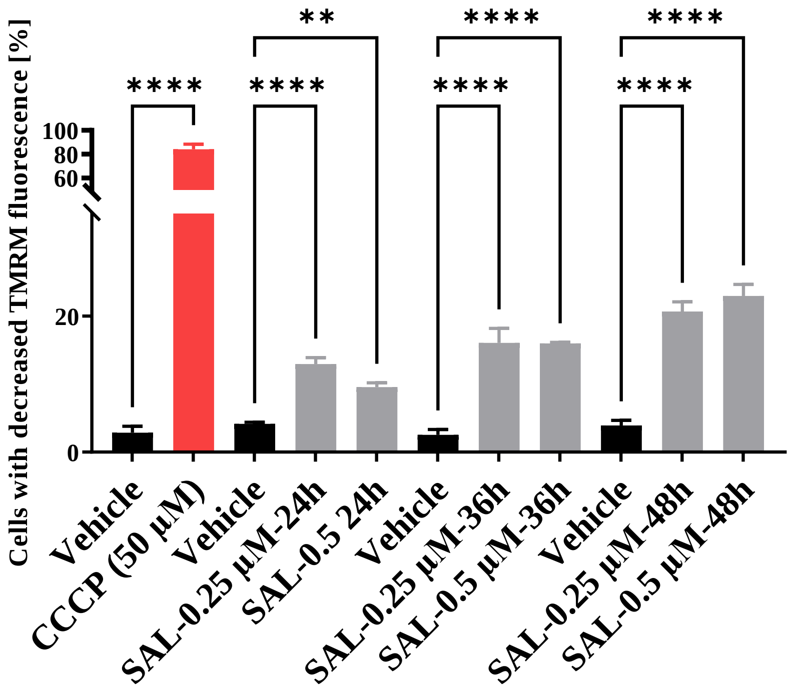

Supplementary Figure S2: Time-course analysis of SAL-induced changes in MMP using TMRM staining.

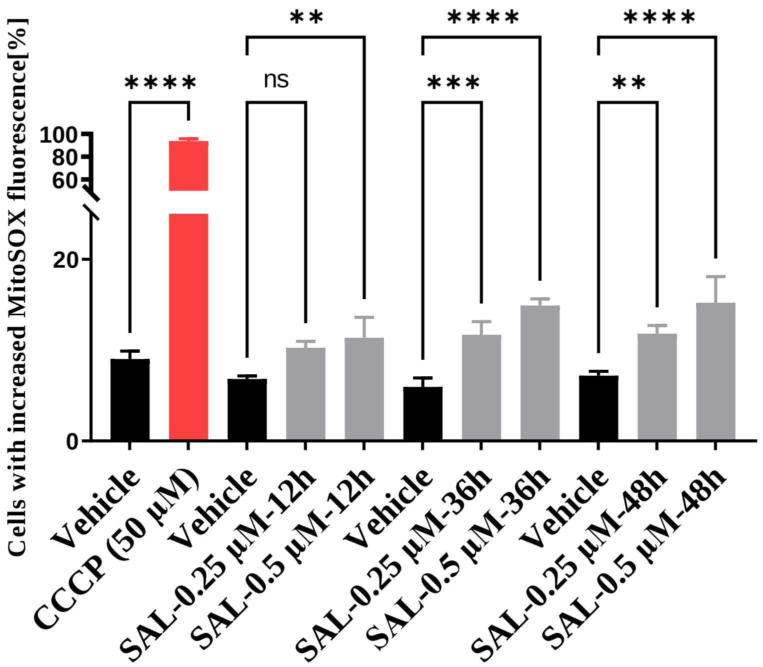

Supplementary Figure S3: Monitoring mitochondrial superoxide levels over time following SAL treatment using MitoSOX.

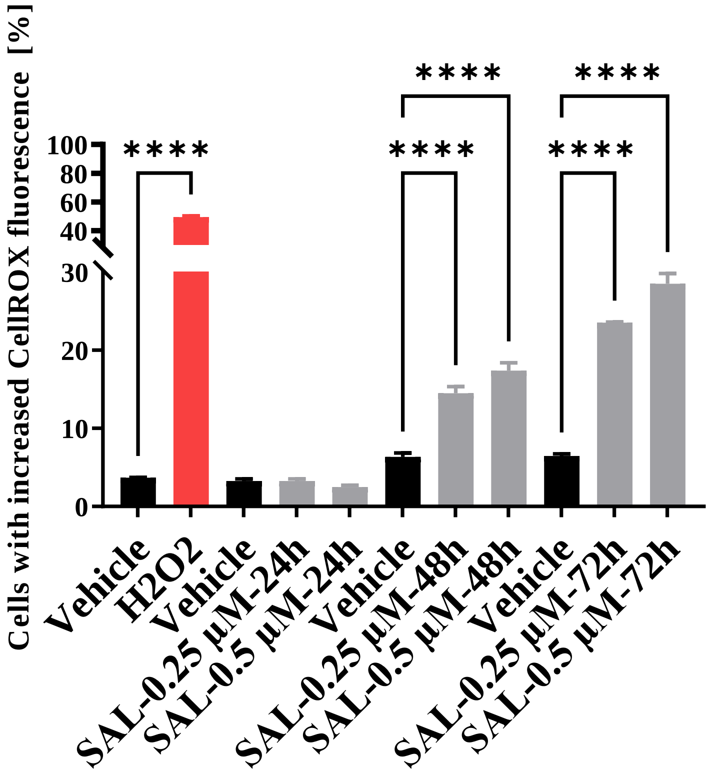

Supplementary Figure S4: Time-dependent changes in cellular oxidative stress in response to SAL treatment, detected via CellROX.

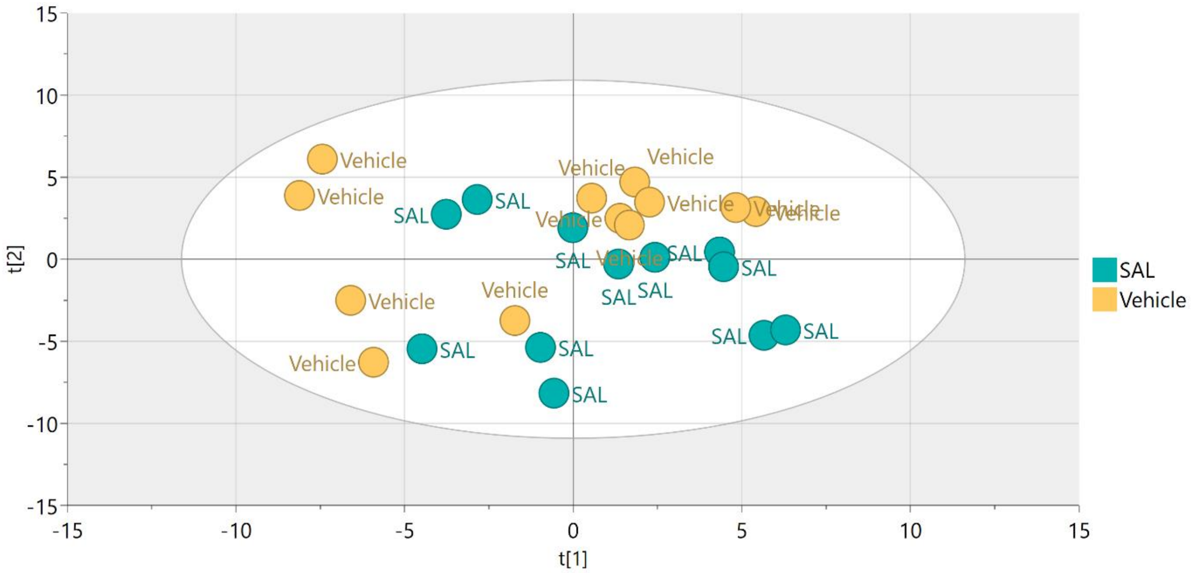

Supplementary Figure S5: Untargeted metabolomic profiling – Principal Component Analysis (PCA) plot.

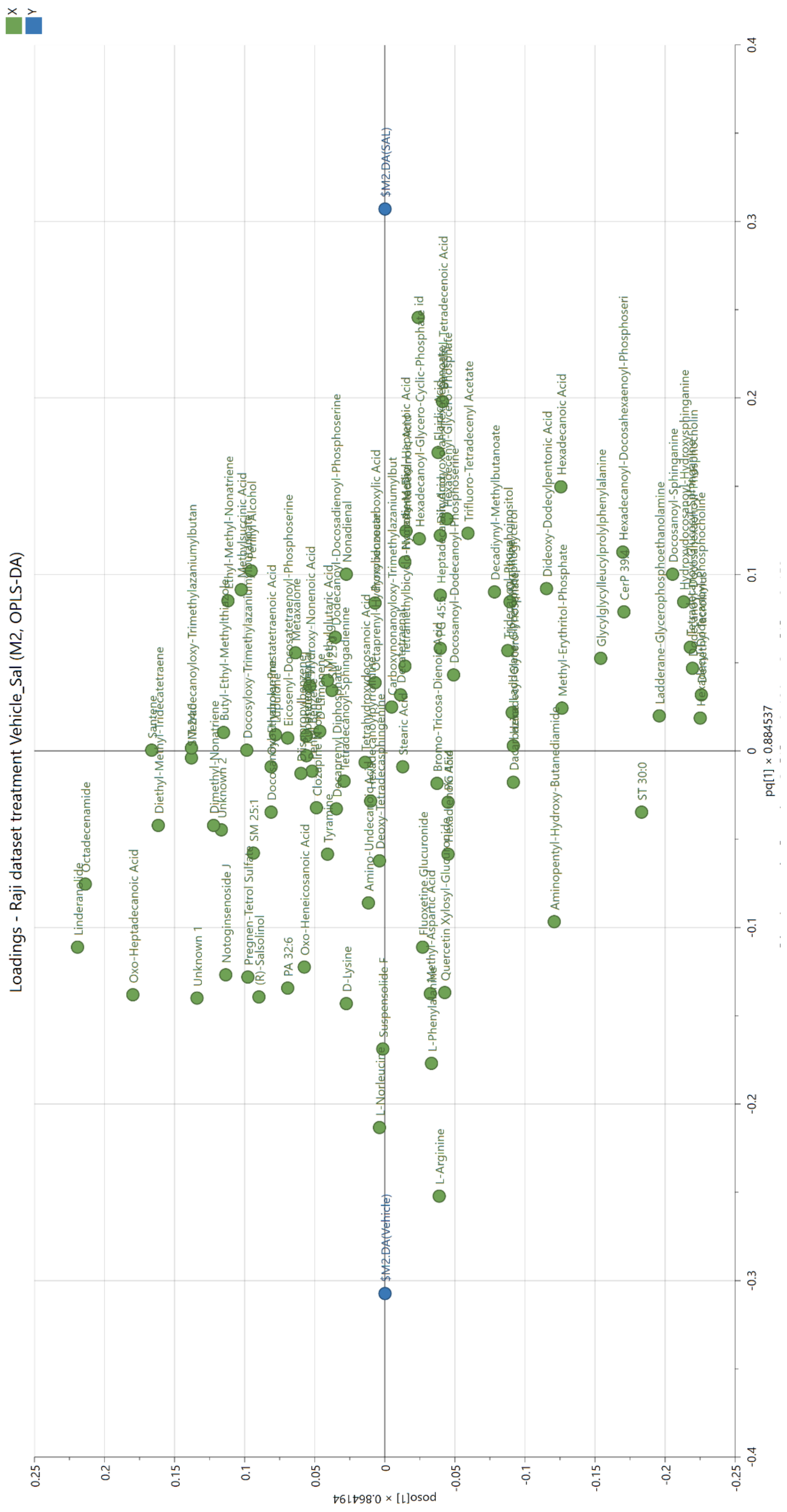

Supplementary Figure S6: Untargeted metabolomic profiling – Orthogonal Partial Least Squares Discriminant Analysis (OPLS-DA) loadings plot.

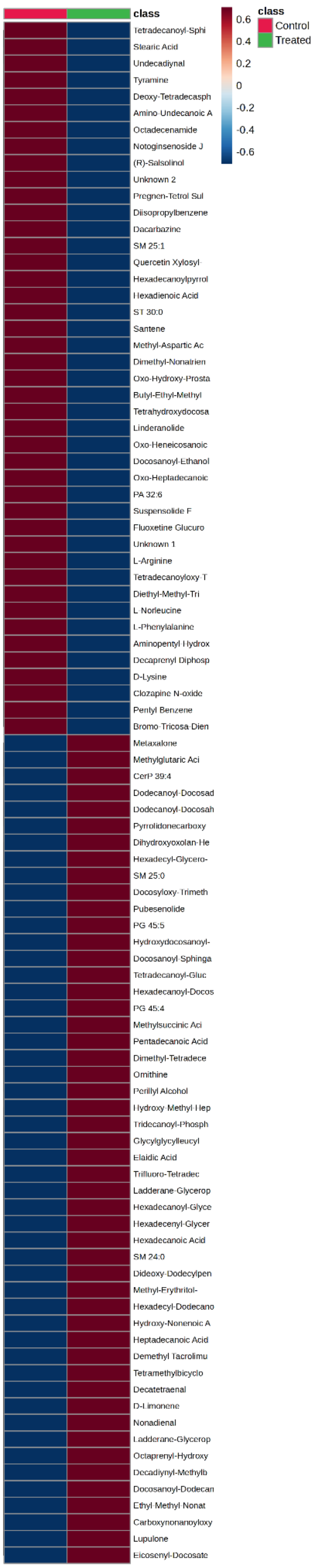

Supplementary Figure S7: Heatmap of metabolite expression patterns from untargeted metabolomic profiling, generated using MetaboAnalyst 6.0.
